# Performance and learning rate prediction models development in FLS and RAS surgical tasks using electroencephalogram and eye gaze data and machine learning

**DOI:** 10.1007/s00464-023-10409-y

**Published:** 2023-09-20

**Authors:** Somayeh B. Shafiei, Saeed Shadpour, Xavier Intes, Rahul Rahul, Mehdi Seilanian Toussi, Ambreen Shafqat

**Affiliations:** 1 Intelligent Cancer Care Laboratory, Department of Urology, Roswell Park Comprehensive Cancer Center, Buffalo, NY 14263 USA; 2 University of Guelph, Guelph, ON N1G 2W1 Canada; 3 Rensselaer Polytechnic Institute, 110 8th Street, Troy, NY 12180 USA

**Keywords:** Peg transfer, Pattern cut, Suturing, Tissue dissection

## Abstract

**Objective:**

This study explored the use of electroencephalogram (EEG) and eye gaze features, experience-related features, and machine learning to evaluate performance and learning rates in fundamentals of laparoscopic surgery (FLS) and robotic-assisted surgery (RAS).

**Methods:**

EEG and eye-tracking data were collected from 25 participants performing three FLS and 22 participants performing two RAS tasks. Generalized linear mixed models, using L1-penalized estimation, were developed to objectify performance evaluation using EEG and eye gaze features, and linear models were developed to objectify learning rate evaluation using these features and performance scores at the first attempt. Experience metrics were added to evaluate their role in learning robotic surgery. The differences in performance across experience levels were tested using analysis of variance.

**Results:**

EEG and eye gaze features and experience-related features were important for evaluating performance in FLS and RAS tasks with reasonable results. Residents outperformed faculty in FLS peg transfer (*p* value = 0.04), while faculty and residents both excelled over pre-medical students in the FLS pattern cut (*p* value = 0.01 and *p* value < 0.001, respectively). Fellows outperformed pre-medical students in FLS suturing (*p* value = 0.01). In RAS tasks, both faculty and fellows surpassed pre-medical students (*p* values for the RAS pattern cut were 0.001 for faculty and 0.003 for fellows, while for RAS tissue dissection, the *p* value was less than 0.001 for both groups), with residents also showing superior skills in tissue dissection (*p* value  = 0.03).

**Conclusion:**

Findings could be used to develop training interventions for improving surgical skills and have implications for understanding motor learning and designing interventions to enhance learning outcomes.

**Graphical abstract:**

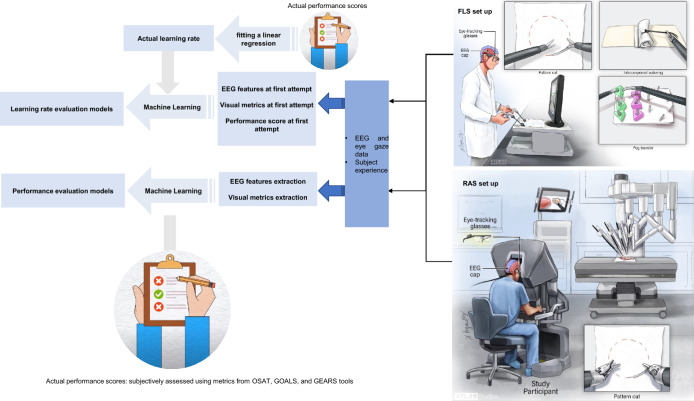

**Supplementary Information:**

The online version contains supplementary material available at 10.1007/s00464-023-10409-y.

Surgery has evolved with minimally invasive techniques like laparoscopic surgery gaining popularity due to several advantages over traditional open surgery, including smaller incisions, reduced postoperative pain, faster recovery, and improved cosmetic outcomes [1, 2]. However, laparoscopic surgery requires specialized skills and techniques that differ from those used in open surgery [3].

The FLS program trains and assesses necessary skills for safe and effective laparoscopic surgery through simulated tasks [3]. The program evaluates important elements, such as expertise, decision-making abilities, and manual capabilities, to determine laparoscopic surgical competence [4].

RAS, on the other hand, uses robotic technology to assist surgeons in performing intricate surgical procedures. RAS is now commonly used in various surgical specialties, such as urology, gynecology, and general surgery. Mastering the FLS is crucial for surgical training and is an essential prerequisite for performing RAS surgeries.

Participation in the FLS program improves surgical trainees’ technical skills [5]. Additionally, completion of the FLS program is often a requirement for board certification in surgical specialties [5]. Hence, FLS tasks are critical components of laparoscopic and RAS surgical training.

Evaluating performance in FLS tasks can be challenging because (1) FLS score heavily weighs time and precision in its formula [6], (2) subjective assessment of task performance can also be challenging due to variations in criteria used by different observers, (3) individual differences in cognitive and physical abilities, along with external factors like fatigue and stress, can also make it challenging to assess performance accurately [7].

Recent technological advancements have integrated physiological and cognitive measures to enhance surgical task performance evaluation. EEG and eye tracking features are two methods that have gained attention recently. EEG measures electrical activity in the brain and can assess cognitive processing. Studies indicate that EEG features, such as event-related potentials, decrease at the parietal electrode with skill acquisition in laparoscopic surgery [8]. Eye tracking features, such as gaze patterns and fixations, can reveal visual attention and decision-making insights [9]. Expert laparoscopic surgeons exhibit shorter fixations and longer saccades compared to novices, indicating more efficient visual search and decision-making [10]. Eye tracking was suggested as a potential surgical skill evaluation tool [10, 11].

This study developed models for evaluating performance and learning rate in FLS and RAS tasks using machine learning, EEG, and eye gaze features.

## Methods

This study was approved by the Institutional Review Board (IRB: I-241913) of Roswell Park Comprehensive Cancer Center. The IRB granted permission to waive the need for written consent. Participants were given written information about the study and provided verbal consent.

### Data recording

**Fig. 1:**
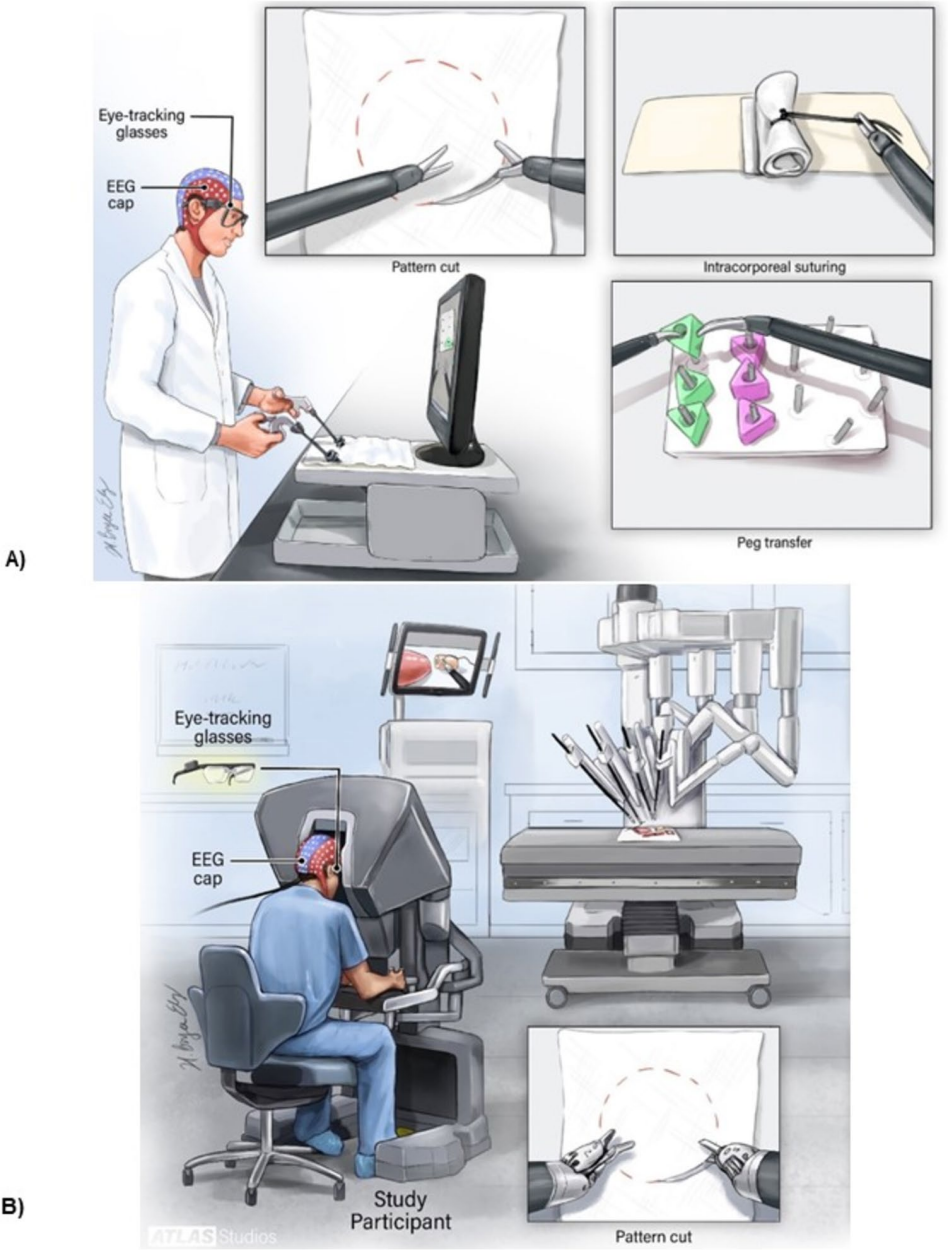
Experimental set up. Representation of participant performing FLS tasks on laparoscopic training box (**A**) and RAS tasks on the da Vinci robot (**B**) while wearing EEG headcap and Tobii eyeglasses

A 124-channel EEG headset (AntNeuro®) was used to record EEG data at 500 Hz (Fig. 1). Additionally, Tobii eyeglasses (Tobii®) were used to simultaneously record eye-tracking data at 50 Hz (Fig. 1). Videos were also recorded during the task completion.

### Participants

Eleven medical or premedical students, two residents, six fellows, and six surgeons participated in this study. The participants’ ages ranged from 22 to 67, with an average age of 36 ± 12. There were 17 male and 8 female participants, of whom 24 were right-handed and one was left-handed. Additionally, 17 participants were right eye dominant, while 8 were left eye dominant. Three participants did not perform RAS tasks. The number of hours of RAS experience, the total count of laparoscopic surgeries performed as the primary surgeon (cases), the length of clinical practice (years), and the duration of formal laparoscopic surgery training (years) for participants were represented in Table 1.

**Table 1:** Representation of participants’ experience in laparoscopic surgery and robot-assisted surgery

Participant	Hours of RAS experience	Number of laparoscopic surgeries as the primary surgeon (cases)	Years of clinical practice	Years of formal training in laparoscopic surgery	Experience level
1	500	0	0	0	Fellow
2	100	50	0	0	Fellow
3	0	0	0	0	Pre-medical student
4	0	0	0	0	Pre-medical student
5	120	75	0	5	Fellow
6	100	0	0	0	Fellow
7	10	25	0	0	Resident
8	30	250	10	0	Faculty
9	500	250	10	1	Faculty
10	0	0	0	0	Pre-medical student
11	1000	250	7	5	Faculty
12	1000	0	10	0	Faculty
13	0	0	0	0	Pre-medical student
14	1000	75	2	5	Faculty
15	0	0	0	0	Pre-medical student
16	0	0	0	0	Pre-medical student
17	0	0	0	0	Pre-medical student
18	0	0	0	0	Pre-medical student
19	15	0	0	3.5	Fellow
20	0	0	0	0	Pre-medical student
21	0	0	0	0	Pre-medical student
22	40	25	0	2	Resident
23	55	0	7	0	Faculty
24	0	0	0	0	Pre-medical student
25	0	0	0	3.5	Fellow

### Tasks

The study comprised three FLS program tasks (peg transfer, pattern cut, and intracorporeal suturing) and two RAS tasks (pattern cut and tissue dissection). Participants performed each task five times, while expert surgeons only completed them twice. FLS tasks were done with the FLS laparoscopic training box (Pyxus®), and RAS tasks were performed using the da Vinci surgical robot (Fig. 1).

FLS peg transfer involves participants transferring six objects mid-air from their non-dominant hand to their dominant hand and placing them on a peg on the opposite side of the pegboard. They then reverse the process, transferring the objects back to their original side. Dropping objects outside the field of view incurs a penalty. It evaluates a surgeon’s fine motor skills, hand–eye coordination, and depth perception [12].

FLS pattern cut involves holding a Maryland dissector in one hand and providing traction to a gauze piece while cutting it with endoscopic scissors held in the other hand. Participants cut along a pre-marked circle until the gauze is completely removed from the 4 × 4 gauze piece. Any cuts deviating from the marked circle are penalized. This task assesses skills needed for laparoscopic surgery, including hand–eye coordination, dexterity, and depth perception [12].

FLS intracorporeal suturing involves placing a short suture through two marks in a Penrose drain and tying two throws of a knot to close a slit. Penalties are assessed for deviations from the marks, improper closure of the slit, or a knot that slips or comes apart when tension is applied. This task evaluates surgical skills, including hand–eye coordination, dexterity, knot-tying in tight spaces, tissue handling, tension management, and suturing techniques [13].

RAS pattern cut and tissue dissection involve cutting along a pre-marked circle on paper and woodchuck skin, respectively, until the circle area is completely removed.

### Actual performance scores

FLS peg transfer performance was evaluated by counting completion time, tool collisions, and drops from videos, and using the Global Operative Assessment of Laparoscopic Skills (GOALS) tool to evaluate five domains (depth perception, bimanual dexterity, efficiency, tissue handling, and autonomy) on a 1–5 Likert scale, with a total range of 5–25 (“Appendix 1”) [3]. In FLS pattern cut, completion time and tool collisions were counted via videos, error area was calculated using the final product and Fiji software [14], and used to assess overall technical proficiency using the GOALS tool. In FLS suturing, videos were used to count time to complete the task, number of drops and collisions, and evaluated performance using the Objective Structured Assessment of Technical Skills (OSAT) tool, which assesses eight domains (respect for tissues, time and motion, instrument handling, suture handling, flow of suturing, knowledge of the steps, overall appearance, and overall performance domains) on a Likert scale between 1 and 5, with a total score range of 8–40 (“Appendix 1”) [15].

For RAS pattern cut and tissue dissection, completion time, tool collisions, and error area were counted using videos and Fiji software [14], respectively. Performance was assessed using the Global Evaluative Assessment of Robotic Skills (GEARS) tool [16], which measures depth perception, bimanual dexterity, efficiency, force sensitivity, autonomy, and robotic control on a 1–5 Likert scale (“Appendix 1”).

### Actual learning rate

The learning rate was calculated by fitting a linear regression to the participant’s performance scores across attempts and taking the slope of the resulting line.

### Eye gaze features

Eye gaze data collected in this study were preprocessed using Tobii Pro Lab©. Preprocessing involved applying a moving average filter with a window size of 3 points to reduce noise, and a velocity-threshold identification fixation filter with a threshold of 30° per second to identify fixation and saccadic points. Twelve eye gaze features were extracted, including pupil diameter, entropy, fixation time points, saccade time points, gaze direction change, and pupil trajectory length for both eyes [17, 18].

### EEG features

Signal processing techniques were applied to the EEG signals to remove artifacts (Supplement 1 [19–35]). After decontamination, the EEG signals were analyzed to extract features related to changes of brain activity during learning, such as strength, search information, temporal network flexibility, integration, and recruitment (Supplement 1). The average of features was calculated at 4 different cortices of the brain (frontal, parietal, occipital, and temporal cortices), resulting in 20 EEG features.

### Role of extracted EEG features in learning

When a person learns new skills, brain stores information in particular areas [36]. The process of practicing and training results in modifications to the brain’s functional network [36], which can be measured by examining various features, such as strength, search information, temporal network flexibility, integration, and recruitment. Search information measures the efficiency of information transfer between different areas of the brain [24, 37]. Strength measures the quality of communication between different regions of the brain. Temporal network flexibility measures the degree of the brain changes over time to adapt to different demands [33]. Flexibility has been proposed as a functional brain network feature that changes by learning [38], and as a predictor of the mental workload of surgeons during surgical procedures [39]. Integration explains how different regions of the brain function in harmony over time [34]. Recruitment is the activation of a specific region of the brain that forms interconnected networks while performing cognitive or behavioral tasks. This feature provides insights into the underlying neural mechanisms of different cognitive functions and can assist understand how the brain processes information and generates behavior [40]. Integration and recruitment features are known to be sensitive to changes in skill level and learning [34].

### Performance and learning rate evaluation models using EEG and eye gaze features, and experience

Experience-related features were added to EEG and eye gaze features to explore the influence of RAS and FLS experience on performance and learning rate. These variables included the number of hours of RAS experience, the total count of laparoscopic surgeries performed as the primary surgeon (cases), the length of clinical practice (years), and the duration of formal laparoscopic surgery training (years).

Machine learning models were developed for performance, using retrieved EEG and eye gaze features, and experience-related features. Also, the retrieved EEG and eye gaze features at the first attempt were used to develop learning rate evaluation models. Moreover, performance score at the first attempt of each task was considered as a baseline and was included as a possible predictor of learning rate.

### Machine learning models for performance and learning rate evaluation

The generalized linear mixed models (GLMMs), using L1-penalized estimation, also known as GLMM-LASSO models were developed to select the most important features and evaluate performance. The algorithm was applied to the features with participant identifier (ID) as a random effect, and the best penalty values were selected based on grid search and cross-validation analyses to determine the optimum lambda value with minimum Bayesian Information Criterion (BIC)—Supplement 2.

Learning rate models were developed using EEG and eye gaze features and performance, at the first attempt, experience-related features, and linear regression algorithm. Feed forward features selection and leave one out cross validation techniques were used to select features for linear regression model development.

Local Outlier Factor (LOF) algorithm with 10 neighbors was applied to detect and exclude outliers from analysis. The *R*^2^ metric measured the proportion of variance in the dependent variable explained by the independent variables in the developed models. Mean Absolute Error (MAE) is the average of the absolute differences between the predicted and actual values. Root Mean Squared Error (RMSE) is the square root of the average of the squared differences between the predicted and actual values. MAE and RMSE are two commonly used metrics in machine learning and data science for evaluating the performance of regression models. *R*^2^, MAE, and RMSE metrics were calculated to assess the power of prediction models.

### Statistical analysis to find the change in performance across experience levels

A linear mixed model was fitted for performance scores, where the skill levels were treated as four factors (pre-medical student, resident, fellow, and faculty), and participant Identifier (ID) was treated as a random effect to accommodate for repeated measurement. Analysis of variance (ANOVA) was fitted to test whether there was any difference in measurements between different skill levels. A *p* value less than 0.05 was considered a statistically significant difference between skill levels. Least Squares Means (LSM) was calculated for each skill level to accommodate the inferential comparison.

## Results

The results of this study included the development of performance and learning rate evaluation models, employing EEG and eye gaze features along with experience-related features. The developed models were shown across several tables: Table 2 presented the evaluation model for the FLS peg transfer task; Table 3 showed the models for the FLS pattern cut task; Table 4 outlined the models for the FLS suturing task; Tables 5 and 6 respectively presented the performance and learning rate evaluation models for the RAS pattern cut task; and Tables 7 and 8 respectively depicted the models for the RAS tissue dissection task.

**Table 2:** Performance and learning rate evaluation models at FLS peg transfer using EEG and eye gaze features, and experience-related features

Predictors of performance, GLMM-LASSO model^a^	Estimates	Standard error	*p* value
Average pupil diameter, nondominant eye	1.73	1.14	0.13
Average pupil diameter, dominant eye	2.29	1.14	**0.04**
Entropy of pupil diameter, nondominant eye	− 0.42	0.51	0.41
Entropy of pupil diameter, dominant eye	− 1.18	0.50	**0.018**
Rate of gaze direction change, dominant eye, the horizontal direction	− 0.11	0.39	0.78
Rate of gaze direction change, dominant eye, the vertical direction	0.34	0.25	0.17
Average temporal network flexibility of channels in the parietal cortex	0.03	0.24	0.89
Average recruitment of channels in the parietal cortex	1.68	0.58	**0.004**
Average search information for channels in the parietal cortex	− 1.27	0.63	**0.04**
Average strength of channels in the parietal cortex	− 0.85	0.77	0.27
Average recruitment of channels in the frontal cortex	− 0.49	0.52	0.34
Average search information for channels in the frontal cortex	− 0.29	0.83	0.73
Average strength of channels in the frontal cortex	1.18	0.76	0.12
Average temporal network flexibility of channels in the occipital cortex	− 0.38	0.18	**0.04**
Average integration between channels in the occipital cortex and channels from other cortices	− 0.17	0.42	0.68
Average recruitment of channels in the occipital cortex	0.19	0.52	0.71
Average search information for channels in the occipital cortex	0.75	0.78	0.34
Average strength of channels in the occipital cortex	− 0.04	0.66	0.95
Average search information for channels in the temporal cortex	0.63	0.87	0.46
Average strength of channels in the temporal cortex	0.05	1.09	0.96
Hours of RAS experience	− 1.10	0.60	0.07
Number of laparoscopic surgeries as the primary surgeon (cases)	1.15	0.74	0.12
Years of clinical practice	− 0.38	0.80	0.63
Years of formal training in laparoscopic surgery	− 0.10	0.66	0.87

Predictors of learning rate, feed-forward linear regression model^b^	Estimates	Confidence interval	*p* value
Average pupil diameter, dominant eye	− 0.56	− 0.75 to − 0.37	**< 0.001**
Rate of gaze direction change, dominant eye, the horizontal direction	0.45	0.26 to 0.65	**< 0.001**
Hours of RAS experience	0.46	0.25 to 0.67	**< 0.001**
Average temporal network flexibility of channels in the frontal cortex	− 0.33	− 0.51 to − 0.15	**0.001**
Number of laparoscopic surgeries as the primary surgeon (cases)	0.46	0.18 to 0.73	**0.003**
Years of clinical practice	− 0.32	− 0.60 to − 0.04	**0.029**

Statistically significant values are given in bold (*p* < 0.05)

^a^MAE = 0.80; RMSE = 1.02; * R*^2^ = 0.87

^b^Observations: 25; MAE: 0.25; RMSE: 0.32; * R*^2^: 0.88

**Table 3:** Performance and learning rate evaluation models at FLS pattern cut using EEG and eye gaze features, and experience-related features

Predictors of performance, GLMM-LASSO model^a^	Estimates	Standard error	*p* value
Entropy of pupil diameter, nondominant eye	− 0.14	0.41	0.73
Rate of gaze direction change, nondominant eye, the horizontal direction	0.94	0.32	**0.004**
Average integration between channels in the parietal cortex and channels in other cortices	− 0.38	0.76	0.61
Average search information for channels in the parietal cortex	− 0.29	0.66	0.66
Average strength of channels in the parietal cortex	− 0.86	0.47	0.06
Average integration between channels in the frontal cortex and channels in other cortices	− 0.16	0.79	0.84
Average recruitment of channels in the frontal cortex	− 0.14	0.4	0.72
Average temporal network flexibility of channels in the occipital cortex	0.05	0.13	0.69
Average strength of channels in the occipital cortex	1.05	0.48	**0.03**
Average search information for channels in the temporal cortex	− 0.05	0.66	0.94
Years of clinical practice	− 0.19	0.42	0.65
Years of formal training in laparoscopic surgery	1.11	0.4	**0.005**

Predictors of learning rate, feed-forward linear regression model^b^	Estimates	Confidence interval	*p* value
Performance at first attempt	− 0.73	− 0.96 to − 0.49	** < 0.001**
Rate of fixation time points	0.24	0.08 to 0.41	**0.006**
Rate of gaze direction change, nondominant eye, the horizontal direction	0.28	0.10 to 0.45	**0.004**
Length of the eye trajectory, nondominant eye	− 0.25	− 0.46 to − 0.04	**0.02**
Average strength of channels in the parietal cortex	− 0.29	− 0.53 to − 0.04	**0.02**
Average search information for channels in frontal cortex	− 0.23	− 0.42 to − 0.04	**0.02**
Years of clinical practice	− 0.18	−0.35 to − 0.01	**0.04**

Statistically significant values are given in bold (*p* < 0.05)

^a^MAE = 0.64; RMSE = 0.79; * R*^2^ = 0.86

^b^Observations: 25; MAE: 0.24; RMSE: 0.27; * R*^2^: 0.82

**Table 4:** Performance and learning rate evaluation models at FLS suturing using EEG and eye gaze features, and experience-related features

Predictors of performance, GLMM-LASSO model^a^	Estimates	Standard error	*p* value
Average pupil diameter, nondominant eye	− 1.86	0.51	**< 0.001**
Entropy of pupil diameter, nondominant eye	− 10.16	2.28	**< 0.001**
Entropy of pupil diameter, dominant eye	− 4.79	0.62	**< 0.001**
Rate of fixation time points	− 1.24	0.30	**< 0.001**
Rate of gaze direction change, nondominant eye, the horizontal direction	0.70	2.26	0.75
Rate of gaze direction change, nondominant eye, the vertical direction	8.79	2.68	** < 0.001**
Rate of gaze direction change, dominant eye, the horizontal direction	1.02	0.47	**0.03**
Rate of gaze direction change, dominant eye, the vertical direction	− 0.22	0.31	0.46
Length of the eye trajectory, dominant eye	1.27	2.42	0.59
Average recruitment of channels in the parietal cortex	− 3.11	0.89	**< 0.001**
Average search information for channels in the parietal cortex	− 0.77	1.04	0.45
Average strength of channels in the parietal cortex	− 4.05	0.87	**< 0.001**
Average temporal network flexibility of channels in the frontal cortex	0.06	0.38	0.87
Average integration between channels in the frontal cortex and channels in other cortices	− 2.38	1.18	**0.04**
Average recruitment of channels in the frontal cortex	3.56	1.48	**0.01**
Average search information for channels in the frontal cortex	− 2.68	1.17	**0.02**
Average strength of channels in the frontal cortex	3.58	0.80	**< 0.001**
Average temporal network flexibility of channels in the occipital cortex	− 0.004	0.19	0.98
Average recruitment of channels in the occipital cortex	− 0.80	0.81	0.32
Average search information for channels in the occipital cortex	6.16	1.06	**< 0.001**
Average recruitment of channels in the temporal cortex	1.19	1.51	0.43
Average search information for channels in the temporal cortex	− 3.31	1.14	**0.003**
Number of laparoscopic surgeries as the primary surgeon (cases)	− 4.07	3.77	0.28
Years of clinical practice	2.23	3.42	0.51
Years of formal training in laparoscopic surgery	5.02	2.96	0.09

Predictors of learning rate, feed-forward linear regression model^b^	Estimates	Confidence interval	*p* value
Average pupil diameter, dominant eye	0.93	0.32 to 1.55	**0.007**
Entropy of pupil diameter, dominant eye	0.88	0.34 to 1.42	**< 0.001**
Average integration between channels in the parietal cortex and channels in other cortices	1.7	0.36 to 3.05	**0.017**
Average integration between channels in the frontal cortex and channels in other cortices	− 2.55	− 4.04 to − 1.05	**0.003**
Average recruitment of channels in the frontal cortex	− 1.26	− 2.07 to − 0.45	**0.005**
Average integration between channels in the occipital cortex and channels from other cortices	− 3.89	− 5.62 to − 2.17	**0.001**
Average integration between channels in the temporal cortex and channels from other cortices	3.71	0.84 to 6.58	**0.016**
Years of clinical practice	1.13	0.32 to 1.93	**0.01**
Average strength of channels in the temporal cortex	− 0.68	− 1.31 to − 0.05	**0.037**
Hours of RAS experience	− 1	1.74 to − 0.26	**0.012**

Statistically significant values are given in bold (*p* < 0.05)

^a^MAE = 1.47; RMSE = 1.89; *R*^2^ = 0.92

^b^Observations: 24; MAE: 0.57; RMSE: 0.67; * R*^2^: 0.87

**Table 5:** Performance evaluation models at RAS pattern cut using EEG and eye gaze features, and experience-related features

Predictors of performance, GLMM-LASSO model	Estimates	Standard error	*p* value
Average pupil diameter, nondominant eye	− 1.54	1.07	0.15
Average pupil diameter, dominant eye	− 1.40	1.12	0.21
Entropy of pupil diameter, nondominant eye	0.18	0.74	0.80
Entropy of pupil diameter, dominant eye	0.21	0.20	0.29
Rate of fixation time points	− 0.29	0.24	0.23
Rate of saccade time points	0.10	0.23	0.66
Rate of gaze direction change, nondominant eye, the horizontal direction	1.02	1.06	0.33
Rate of gaze direction change, nondominant eye, the vertical direction	− 1.13	0.54	**0.03**
Rate of gaze direction change, dominant eye, the vertical direction	0.24	0.21	0.23
Length of the eye trajectory, nondominant eye	0.20	0.23	0.38
Length of the eye trajectory, dominant eye	0.04	0.87	0.95
Average temporal network flexibility of channels in the parietal cortex	− 0.37	0.25	0.14
Average integration between channels in the parietal cortex and channels in other cortices	2.93	2.16	0.17
Average recruitment of channels in the parietal cortex	− 0.03	0.86	0.97
Average search information for channels in the parietal cortex	− 0.29	0.61	0.62
Average strength of channels in the parietal cortex	1.39	0.62	**0.02**
Average temporal network flexibility of channels in the frontal cortex	− 0.35	0.20	0.08
Average integration between channels in the frontal cortex and channels in other cortices	− 0.67	1.67	0.68
Average recruitment of channels in the frontal cortex	− 1.52	0.44	** < 0.001**
Average search information for channels in the frontal cortex	0.17	0.57	0.77
Average strength of channels in the frontal cortex	− 0.62	0.61	0.31
Average temporal network flexibility of channels in the occipital cortex	0.26	0.18	0.15
Average integration between channels in the occipital cortex and channels from other cortices	2.23	1.10	**0.04**
Average search information for channels in the occipital cortex	− 0.20	0.43	0.63
Average strength of channels in the occipital cortex	− 0.15	0.56	0.77
Average temporal network flexibility of channels in the temporal cortex	− 0.15	0.28	0.59
Average integration between channels in the temporal cortex and channels from other cortices	− 4.36	2.24	0.05
Average recruitment of channels in the temporal cortex	− 0.33	0.80	0.67
Average search information for channels in the temporal cortex	0.39	0.63	0.53
Average strength of channels in the temporal cortex	− 0.55	0.84	0.51
Hours of RAS experience	2.43	1.28	0.05
Number of laparoscopic surgeries as the primary surgeon (cases)	0.26	1.42	0.85
Years of clinical practice	− 1.24	1.40	0.37
Years of formal training in laparoscopic surgery	1.29	1.19	0.27

Statistically significant values are given in bold (*p* < 0.05)

MAE = 0.74; RMSE = 0.97; *R*^2^ = 0.94

**Table 6:** Learning rate evaluation models at RAS pattern cut using EEG and eye gaze features, and experience-related features

Predictors of learning rate, Feed-forward linear regression model	Estimates	Confidence interval	*p* value
Entropy of pupil diameter, nondominant eye	0.33	0.19 to 0.47	**< 0.001**
Length of the eye trajectory, dominant eye	− 0.24	− 0.42 to − 0.07	**0.01**
Average integration between channels in the parietal cortex and channels in other cortices	0.31	0.13 to 0.49	**0.003**
Average strength of channels in the parietal cortex	− 0.16	− 0.31 to − 0.01	**0.039**
Average recruitment of channels in frontal cortex	− 0.25	− 0.42 to − 0.08	**0.008**
Years of formal training in laparoscopic surgery	− 0.21	− 0.39 to − 0.03	**0.024**

Statistically significant values are given in bold (*p* < 0.05)

Observations: 21; MAE: 0.15; RMSE: 0.21; *R*^2^: 0.84

**Table 7:** Performance evaluation models at RAS tissue dissection using EEG and eye gaze features, experience

Predictors of performance, GLMM-LASSO model	Estimates	Standard error	*p* value
Average pupil diameter, nondominant eye	− 1.23	0.54	**0.02**
Entropy of pupil diameter, nondominant eye	− 4.87	1.34	** < 0.001**
Entropy of pupil diameter, dominant eye	0.27	0.35	0.43
Rate of fixation time points	0.04	0.32	0.90
Rate of saccade time points	0.13	0.35	0.70
Rate of gaze direction change, nondominant eye, the horizontal direction	2.28	0.84	**0.007**
Rate of gaze direction change, nondominant eye, the vertical direction	4.32	1.50	**0.004**
Rate of gaze direction change, dominant eye, the horizontal direction	− 0.83	0.42	**0.04**
Rate of gaze direction change, dominant eye, the vertical direction	− 1.28	0.67	0.05
Length of the eye trajectory, nondominant eye	− 0.27	0.47	0.56
Average temporal network flexibility of channels in parietal cortex	0.12	0.43	0.78
Average recruitment of channels in parietal cortex	− 0.24	0.78	0.75
Average search information for channels in parietal cortex	0.34	0.72	0.63
Average strength of channels in parietal cortex	− 0.60	0.92	0.51
Average temporal network flexibility of channels in frontal cortex	0.01	0.42	0.97
Average recruitment of channels in frontal cortex	− 0.40	0.59	0.49
Average search information for channels in frontal cortex	− 2.14	0.82	**0.01**
Average strength of channels in frontal cortex	− 0.54	0.67	0.41
Average temporal network flexibility of channels in occipital cortex	0.46	0.51	0.36
Average recruitment of channels in occipital cortex	0.43	0.60	0.46
Average search information for channels in occipital cortex	0.74	0.64	0.24
Average strength of channels in occipital cortex	− 0.74	0.76	0.33
Average temporal network flexibility of channels in temporal cortex	− 0.90	0.61	0.14
Average integration between channels in temporal cortex and channels from other cortices	− 1.06	0.61	0.08
Average search information for channels in temporal cortex	0.32	0.69	0.64
Average strength of channels in temporal cortex	1.90	1.18	0.10
Hours of RAS experience	2.68	1.15	**0.02**
Number of laparoscopic surgeries as the primary surgeon (cases)	− 0.60	1.04	0.56
Years of clinical practice	0.86	1.32	0.51
Years of formal training in laparoscopic surgery	0.52	0.88	0.55

Statistically significant values are given in bold (*p* < 0.05)

MAE = 0.60; RMSE = 0.76; *R*^2^ = 0.97

**Table 8:** Learning rate evaluation models at RAS tissue dissection using EEG and eye gaze features, and experience-related features

Predictors of learning rate, Feed-forward linear regression model	Estimates	Confidence interval	*p* value
Entropy of pupil diameter, nondominant eye	2.82	1.70 to 3.95	**< 0.001**
Rate of gaze direction change, nondominant eye, the vertical direction	− 2.08	− 3.15 to − 1.01	**< 0.001**
Average strength of channels in the frontal cortex	− 1.11	− 1.51 to − 0.72	**< 0.001**
Average integration between channels in the occipital cortex and channels from other cortices	0.95	0.59 to 1.31	**< 0.001**
Average recruitment of channels in the occipital cortex	0.46	0.16 to 0.75	**0.006**
Average recruitment of channels in the temporal cortex	− 0.66	− 0.96 to − 0.35	**< 0.001**
Number of laparoscopic surgeries as the primary surgeon (cases)	0.66	0.24 to 1.08	**0.005**
Years of clinical practice	− 0.75	− 1.21 to − 0.29	**0.004**

Statistically significant values are given in bold (*p* < 0.05)

Observations: 21; MAE: 0.28; RMSE: 0.35; *R*^2^: 0.85

Several EEG features at different brain cortices, and eye gaze features played important roles in performance and learning rate evaluation across all tasks. Experience-related features also emerged as pivotal determinants in evaluating the performance and learning rate. Specifically, hours of RAS experience showed a statistically significant association with learning rate for the FLS peg transfer. Similarly, years of clinical practice was associated with learning rate for FLS suturing. The duration of formal training in laparoscopic surgery had a strong association with the learning rate at the RAS pattern cut. Moreover, the quantity of laparoscopic surgeries where the individual was the primary surgeon demonstrated an association with the learning rate at RAS tissue dissection.

### Change in performance across experience levels

From the comparison of performance across four categories (Faculty, fellow, resident, and pre-medical student), the results varied across different tasks. The results showed no statistically significant differences among the categories in performing FLS peg transfer, with *p* values exceeding the common threshold of 0.05 for statistical significance (Table 9).

**Table 9:** Comparison of performance across four categories (faculty, fellow, resident, and pre-medical student)

	Estimate	*p* value	Categories	LSM	SE
*FLS peg transfer task*					
Faculty versus fellow	− 1.29	0.74	Faculty	12.9	0.92
Faculty versus pre-medical student	1.08	0.77	Fellow	14.2	0.90
Faculty versus resident	− 3.49	0.27	Resident	16.4	1.66
Fellow versus pre-medical student	2.37	0.16			
Fellow versus resident	− 2.19	0.64	Pre-medical student	11.8	0.66
Pre-medical student versus resident	− 4.57	0.07			
*FLS pattern cut*					
Faculty versus fellow	− 0.07	0.99	Faculty	13.8	0.57
Faculty versus pre-medical student	2.41	**0.01**	Fellow	13.9	0.55
Faculty versus resident	− 3.30	**0.04**	Resident	17.1	1.02
Fellow versus pre-medical student	2.47	**0.007**			
Fellow versus resident	− 3.23	0.05	Pre-medical student	11.4	0.40
Pre-medical student versus resident	− 5.70	** < 0.001**			
*FLS suturing*					
Faculty versus fellow	− 3.20	0.69	Faculty	32	2.12
Faculty versus pre-medical student	5.76	0.14	Fellow	35.2	2.07
Faculty versus resident	− 3.99	0.78	Resident	36	3.72
Fellow versus pre-medical student	8.96	**0.01**			
Fellow versus resident	− 0.78	0.99	Pre-medical student	26.2	1.52
Pre-medical student versus resident	− 9.75	0.09			
*RAS pattern cut*					
Faculty versus fellow	0.15	0.99	Faculty	26.20	1.15
Faculty versus pre-medical student	6.30	**0.001**	Fellow	26.10	1.29
Faculty versus resident	− 0.53	0.99	Resident	26.80	2.69
Fellow versus pre-medical student	6.14	**0.003**			
Fellow versus resident	− 0.68	0.99	Pre-medical student	19.90	0.77
Pre-medical student versus resident	− 6.83	0.09			
*RAS tissue dissection*					
Faculty versus fellow	2.72	0.13	Faculty	28.70	0.86
Faculty versus pre-medical student	10.29	** < 0.001**	Fellow	26.0	0.85
Faculty versus resident	4.23	0.22	Resident	24.50	1.98
Fellow versus pre-medical student	7.57	** < 0.001**			
Fellow versus resident	1.51	0.89	Pre-medical student	18.40	0.55
Pre-medical student versus resident	− 6.06	**0.03**			

Statistically significant values are given in bold (*p* < 0.05)

*LSM* least squares means, *SE* standard error

At FLS pattern cut, there were significant differences in the scores of faculties versus pre-medical student (*p* = 0.01), fellow versus pre-medical student (*p* = 0.007), and pre-medical student versus resident (*p* < 0.001), indicating that faculty, fellows, and residents performed this task significantly better than pre-medical students. Fellow versus pre-medical student was the only comparison with a significant difference (*p* = 0.01) in FLS suturing, suggesting fellows performed significantly better in suturing tasks compared to pre-medical students. At RAS pattern cut, significant differences were found in the performance between faculty and pre-medical student (*p* = 0.001) and between fellow and pre-medical student (*p* = 0.003), both indicating superior performance by faculty and fellows compared to pre-medical students. At RAS tissue dissection, the comparisons between faculty and pre-medical student (*p* < 0.001), fellow and pre-medical student (*p* < 0.001), and pre-medical student versus resident (p = 0.03) showed significant differences, suggesting that faculty, fellows, and residents performed better at RAS tissue dissection than pre-medical students.

## Discussion

Better methods for performance and learning rate evaluation are necessary to improve surgical training while ensuring patient safety. The best existing performance evaluation approaches are based on subjective rating scales, which are costly and subject to bias [3]. Objective evaluation methods are needed to enable individualized skill development, which ultimately improves surgical outcomes.

Results indicated that eye movement measures and specific neural activity patterns are significant predictors of performance and learning rate in various surgical tasks. The performance evaluation models demonstrated robust results, as evidenced by the notably high coefficients of determination, or *R*^2^ values, for FLS peg transfer, FLS pattern cut, FLS suturing, RAS pattern cut, and RAS tissue dissection tasks, which were 0.87, 0.86, 0.92, 0.94, and 0.97 respectively. Concurrently, the MAE for these tasks was relatively low, with values of 0.8, 0.64, 1.47, 0.74, and 0.6. Similarly, the learning rate evaluation models showed considerable efficacy, yielding high *R*^2^ values for FLS peg transfer, FLS pattern cut, FLS suturing, RAS pattern cut, and RAS tissue dissection tasks at 0.88, 0.82, 0.87, 0.84, and 0.85, respectively. Correspondingly, the MAE for these tasks was kept low (0.25, 0.24, 0.57, 0.15, and 0.28, respectively). Findings can have important implications for surgical training programs, as they can be tailored to improve these specific aspects of surgeons’ behavior and neural patterns.

The regression analyses provided insight into the roles various features play in determining performance and learning rates in several surgical tasks. Eye-tracking metrics such as pupil diameter and pupil trajectory length were predictors of performance across different surgical tasks, suggesting a link between visual attention and surgical performance. Measures of brain function, including the recruitment and strength of channels in brain cortices, significantly influenced performance. This suggests that neural activity and how the brain processes information during a task could be key indicators of surgical performance. Eye-tracking metrics at the first attempt of each task were significant predictors, implying that initial visual attention and processing may set the rate for learning rate. Similar to performance, brain function features at the first attempt also play a key role in learning rates evaluation. This finding supports the idea that the initial brain-state might shape the trajectory of learning [41].

These findings point to a multifaceted interaction of visual and neurological factors that contribute to surgical performance and the rate of learning surgical tasks. They highlight the potential value of eye-tracking metrics and neuroimaging data in surgical education and training. By understanding these influences, surgical training programs could be tailored to individual learning patterns and optimize performance outcomes. However, further research would be beneficial to confirm these results and develop specific interventions.

The findings emphasize the significance of ocular dominance (dominant and nondominant eyes) in surgical performance, as it plays a crucial role in depth perception and precise manipulation of surgical instruments [42, 43]. However, current assessment tools for surgical skills do not explicitly consider ocular dominance. Therefore, incorporating measurements of ocular dominance could enhance the accuracy of evaluating surgical performance. Surgical training programs that incorporate simulated surgical environments and tools designed to enhance trainees’ ocular dominance and other visual and motor skills could be beneficial.

The number of years of formal training in laparoscopic surgery positively predicted performance in several tasks. This suggests that specific, focused training in a procedure may be important when it comes to skill acquisition and performance in that procedure.

### Change in performance across experience levels

No noticeable statistical differences were observed among the various categories in performing the FLS peg transfer (Table 9). This may be attributed to the straightforward nature of the task, rendering it manageable for all participants. The results indicated a superior performance by the resident group compared to the faculty group in performing FLS pattern cut (*p* value = 0.04). This difference might be attributable to the fact that residents regularly engage in FLS tasks, whereas the faculty members have typically not practiced these tasks since their residency programs, often many years prior. In terms of the FLS pattern cut, the faculty group demonstrated a higher performance level compared to the pre-medical student group (*p* value = 0.01). Similarly, the residents also outperformed the pre-medical students in the same task (*p* value < 0.001). As expected, fellows surpassed pre-medical students in executing the FLS suturing task (*p* value = 0.01).

Regarding RAS tasks, both faculty and fellows exhibited better performances in the RAS pattern cut and RAS tissue dissection tasks compared to the pre-medical students (*p* values for the RAS pattern cut were 0.001 for faculty and 0.003 for fellows, while for RAS tissue dissection, the *p* value was less than 0.001 for both groups). Moreover, residents also demonstrated superior skills in RAS tissue dissection compared to pre-medical students (*p* value = 0.03).

The strengths of this study include its innovative approach of utilizing functional brain network and eye gaze features to assess surgical performance and learning rate. The standardized approach to task selection and data collection enhances the reliability and validity of the findings. Furthermore, by including both FLS and RAS tasks, the study allows for a comparison of performance across different surgical modalities, providing valuable insights. Overall, the study’s methodology, standardized approach, and comparison across surgical modalities contribute to its strengths.

However, several limitations should be considered. The use of linear model analyses in this study does not establish causality. Additionally, the small sample size of 25 participants and five attempts of tasks may limit the generalizability of the results to a broader population. The study focused solely on EEG and eye gaze data, neglecting other factors such as muscle activity that may also influence surgical performance and learning rate. Moreover, the study examined only a limited number of tasks, which may not encompass the full spectrum of surgical procedures. Finally, the controlled laboratory environment may not fully capture the complexity and variability of real-world surgical settings.

#### Practical implications

The developed models for evaluating performance and rate of learning using EEG and eye-tracking characteristics are promising, and they are aligned with the demands of each task. Once these models are validated for a broader population and a variety of surgical procedures, they could be utilized in surgical residency programs to enhance the RAS training process. This can be achieved in two ways: (1) By offering objective, unbiased performance evaluation of RAS trainees without the need for a RAS surgeon present during training sessions. This approach could reduce the costs associated with skill acquisition while offering trainees valuable feedback. This means trainees can correct any errors in their technique rather than repeating them, leading to a faster learning process. This increased efficiency would enable more trainees to enroll in programs, expedite the graduation of current residents, and ultimately increase the number of trained RAS surgeons each year. This proliferation of RAS skills would benefit more patients and hospitals, as RAS procedures are associated with shorter hospital stays and fewer surgical complications than traditional surgical methods [44, 45]. (2) By recording data from the initial attempt, the learning rate evaluation models could assist RAS training programs in predicting an individual trainee’s rate of learning. Equipped with this knowledge, programs could better select trainees or prepare strategies to strengthen learning among slower learners.

Overall, these results suggest that cognitive load, as inferred from eye tracking and EEG data, plays a crucial role in surgical performance and the rate of skill acquisition. This could have several implications for the way surgical training programs are designed: Training could be individualized based on these features, with trainees receiving feedback not only on their technical skills but also on their cognitive load management; Simulators and training programs could incorporate eye tracking and EEG data to provide more detailed feedback; Eye tracking and EEG could be used as objective measures to assess surgical proficiency and readiness for independent practice.

## Conclusion

Results provided valuable insights into the potential for the integration of eye-tracking and neuroimaging measures as objective tools for performance and learning rate evaluation in surgical training. The developed models demonstrate significant potential, as they provide an objective assessment of performance and learning rates. This is an important improvement over more subjective methods, which are costly and susceptible to biases. The results showed that several neural and visual features are meaningful predictors of performance and learning rate in the FLS and RAS surgical tasks. The findings provide insights into the factors that affect task performance and learning rate, which could inform the development of training interventions to improve surgical skill acquisition.

### Electronic supplementary material

Below is the link to the electronic supplementary material.

Supplementary file1 (DOCX 29 kb)

Supplementary file2 (DOCX 330 kb)

## Data Availability

The data analyzed in the current study are available at Shafiei and Shadpour [46]. Integration of electroencephalogram and eye-gaze datasets for performance evaluation in fundamentals of laparoscopic surgery (FLS) tasks (version 1.0.0). PhysioNet.

## Appendix 1: Surgical performance evaluation tools

### Global operative assessment of laparoscopic skills (GOALS) [3]

#### Depth perception

1—Constantly overshoots target, wide swings, slow to correct.

3—Some overshooting or missing target, but quick to correct.

5—Accurately directs instruments in the correct plane to target.

#### Bimanual dexterity

1—Uses only one hand, ignores nondominant hand, poor coordination between hands.

3—Uses both hands, but does not optimize the interaction between hands.

5—Expertly uses both hands in a complementary manner to provide optimal exposure.

#### Efficiency

1—Uncertain, inefficient efforts; many tentative movements; constantly changing focus or persisting without progress.

3—Slow, but planned movements are reasonably organized.

5—Confident, efficient, and safe conduct, maintains focus on the task until it is better performed by way of an alternative approach.

#### Tissue handling

1—Rough movements, tears tissue, injures adjacent structures, poor grasper control, grasper frequently slips.

3—Handles tissue reasonably well, with minor trauma to adjacent tissue (i.e., occasional unnecessary bleeding or slipping of the grasper).

5—Handles tissues well, applies appropriate traction, negligible injury to adjacent structures.

#### Autonomy

1—Unable to complete entire task, even with verbal guidance.

3—Able to complete task safely with moderate guidance.

5—Able to complete task independently without prompting.

### Global evaluative assessment of robotic skills (GEARS) [16]

**Table:**

1	2	3	4	5
Depth perception				
Constantly overshoots target, wide swings, slow to correct		Some overshooting or missing of target, but quick to correct		Accurately directs instruments in correct plane to target
Bimanual dexterity				
Uses only one hand, ignores non-dominant hand, poor coordination		Uses both hands, but does not optimize interactions between hands		Expertly uses both hands in a complementary way to provide best exposure
Efficiency				
Inefficient efforts; many uncertain movements; constantly changing focus or persisting without progress		Slow, but planned movements are reasonably organized		Confident, efficient and safe conduct, maintains focus on task, fluid progression
Force sensitivity				
Rough moves, tears tissue, injures nearby structures, poor control, frequent suture breakage		Handles tissues reasonably well, minor trauma to adjacent tissue, rare suture breakage		Applies appropriate tension, negligible injury to adjacent structures, no suture breakage
Autonomy				
Unable to complete entire task, even with verbal guidance		Able to complete task safely with moderate guidance		Able to complete task independently without prompting
Robotic control				
Consistently does not optimize view, hand position, or repeated collisions even with guidance		View is sometimes not optimal. Occasionally needs to relocate arms. Occasional collisions and obstruction of assistant		Controls camera and hand position optimally and independently. Minimal collisions or obstruction of assistant
Use of third arm: N/A, third arm was not used in this study				
Consistently does not use it, or does not use it well when required, even with verbal guidance		Mostly uses third arm in a safe and efficient manner with moderate guidance		Consistently uses third arm in a safe and efficient manner without prompting

## References

[1] Kim H-H (2010). Morbidity and mortality of laparoscopic gastrectomy versus open gastrectomy for gastric cancer: an interim report—a phase III multicenter, prospective, randomized Trial (KLASS Trial). Ann Surg.

[2] Hwang S-H (2009). Actual 3-year survival after laparoscopy-assisted gastrectomy for gastric cancer. Arch Surg.

[3] Vassiliou MC (2005). A global assessment tool for evaluation of intraoperative laparoscopic skills. Am J Surg.

[4] Peters JH (2004). Development and validation of a comprehensive program of education and assessment of the basic fundamentals of laparoscopic surgery. Surgery.

[5] Sroka G (2010). Fundamentals of laparoscopic surgery simulator training to proficiency improves laparoscopic performance in the operating room—a randomized controlled trial. Am J Surg.

[6] Derossis AM (1998). Development of a model for training and evaluation of laparoscopic skills. Am J Surg.

[7] Kahol K, Vankipuram M, Smith ML (2009). Cognitive simulators for medical education and training. J Biomed Inform.

[8] Thomaschewski M (2021). Changes in attentional resources during the acquisition of laparoscopic surgical skills. BJS Open.

[9] Frutos-Pascual M, Garcia-Zapirain B (2015). Assessing visual attention using eye tracking sensors in intelligent cognitive therapies based on serious games. Sensors.

[10] Kuo R, Chen H-J, Kuo Y-H (2022). The development of an eye movement-based deep learning system for laparoscopic surgical skills assessment. Sci Rep.

[11] Toussi MS (2023). MP26-09 eye movement behavior associates with expertise level in robot-assisted surgery. J Urol.

[12] Emken JL, McDougall EM, Clayman RV (2004). Training and assessment of laparoscopic skills. J Soc Laparoendosc Surg.

[13] Soper NJ, Fried GM (2008). The fundamentals of laparoscopic surgery: its time has come. Bull Am Coll Surg.

[14] Schindelin J (2012). Fiji: an open-source platform for biological-image analysis. Nat Methods.

[15] Alam M (2014). Objective structured assessment of technical skills in elliptical excision repair of senior dermatology residents: a multirater, blinded study of operating room video recordings. JAMA Dermatol.

[16] Goh AC (2012). Global evaluative assessment of robotic skills: validation of a clinical assessment tool to measure robotic surgical skills. J Urol.

[17] Garcia AAT (2021). Biosignal processing and classification using computational learning and intelligence: principles, algorithms, and applications.

[18] Shafiei SB (2023). Developing surgical skill level classification model using visual metrics and a gradient boosting algorithm. Ann Surg Open.

[19] Luck SJ (2014). An introduction to the event-related potential technique.

[20] Kayser J, Tenke CE (2015). On the benefits of using surface Laplacian (current source density) methodology in electrophysiology. Int J Psychophysiol.

[21] Srinivasan R (2007). EEG and MEG coherence: measures of functional connectivity at distinct spatial scales of neocortical dynamics. J Neurosci Methods.

[22] Strotzer M (2009). One century of brain mapping using Brodmann areas. Clin Neuroradiol.

[23] Sneppen K, Trusina A, Rosvall M (2005). Hide-and-seek on complex networks. Europhys Lett.

[24] Rosvall M (2005). Searchability of networks. Phys Rev E.

[25] Trusina A, Rosvall M, Sneppen K (2005). Communication boundaries in networks. Phys Rev Lett.

[26] Lynn CW, Bassett DS (2019). The physics of brain network structure, function and control. Nat Rev Phys.

[27] Zhao H (2022). SCC-MPGCN: self-attention coherence clustering based on multi-pooling graph convolutional network for EEG emotion recognition. J Neural Eng.

[28] Sporns O (2013). Network attributes for segregation and integration in the human brain. Curr Opin Neurobiol.

[29] Betzel RF (2017). Positive affect, surprise, and fatigue are correlates of network flexibility. Sci Rep.

[30] Radicchi F (2004). Defining and identifying communities in networks. Proc Natl Acad Sci USA.

[31] Jutla IS, Jeub LG, Mucha PJ (2011) A generalized Louvain method for community detection implemented in MATLAB. http://netwiki.amath.unc.edu/GenLouvain

[32] Bassett DS (2013). Task-based core-periphery organization of human brain dynamics. PLoS Comput Biol.

[33] Bassett DS (2011). Dynamic reconfiguration of human brain networks during learning. Proc Natl Acad Sci USA.

[34] Bassett DS (2015). Learning-induced autonomy of sensorimotor systems. Nat Neurosci.

[35] Mattar MG (2015). A functional cartography of cognitive systems. PLoS Comput Biol.

[36] Jesan JP, Lauro DM (2003). Human brain and neural network behavior: a comparison. Ubiquity.

[37] Goñi J (2014). Resting-brain functional connectivity predicted by analytic measures of network communication. Proc Natl Acad Sci USA.

[38] Reddy PG (2018). Brain state flexibility accompanies motor-skill acquisition. Neuroimage.

[39] Shafiei SB (2020). Evaluating the mental workload during robot-assisted surgery utilizing network flexibility of human brain. IEEE Access.

[40] Buckner RL, Andrews-Hanna JR, Schacter DL (2008). The brain's default network: anatomy, function, and relevance to disease. Ann NY Acad Sci.

[41] Gabitov E, Manor D, Karni A (2016). Learning from the other limb's experience: sharing the ‘trained’M1 representation of the motor sequence knowledge. J Physiol.

[42] McFadden S, Davies MN, Green PR (1994). Binocular depth perception. Perception and motor control in birds: an ecological approach.

[43] Bogdanova R, Boulanger P, Zheng B (2016). Depth perception of surgeons in minimally invasive surgery. Surgical Innov.

[44] Khorgami Z (2019). The cost of robotics: an analysis of the added costs of robotic-assisted versus laparoscopic surgery using the National Inpatient Sample. Surg Endosc.

[45] Bhama AR (2016). A comparison of laparoscopic and robotic colorectal surgery outcomes using the American College of Surgeons National Surgical Quality Improvement Program (ACS NSQIP) database. Surg Endosc.

[46] Shafiei SB, Shadpour S (2023) Integration of electroencephalogram and eye-gaze datasets for performance evaluation in Fundamentals of Laparoscopic Surgery (FLS) tasks (version 1.0.0). PhysioNet. 10.13026/kyjw-p786.

