# Supplementary material for: Performance and learning rate prediction models development in FLS and RAS surgical tasks using electroencephalogram and eye gaze data and machine learning
Source: Surg Endosc. 2023 Sep 20;37(11):8447–63. doi: 10.1007/s00464-023-10409-y (PMC10615961; doi:10.1007/s00464-023-10409-y)
Supplement: Supplementary file 1 — Supplementary file1 (DOCX 29 kb) [file 464_2023_10409_MOESM1_ESM.docx]

**Supplement 1** EEG data processing and feature extraction

**EEG Pre-processing:** The F8, POz, AF4, AF8, F6, FC3, M1 and M2 (electrodes placed on the mastoids) were excluded in this study due to the poor signal quality. By employing blind source separation and a topographical Principal Component Analysis with the advanced source analysis (ASA) framework created by ANT Neuro Inspiring Technology Inc., Netherlands, the remaining 116 EEG channels were decontaminated from artifacts. Five steps were taken to decontaminate the EEG data: 1) The average of all scalp channels included in the study also known as "common average reference", was used to re-reference the EEG data [19]. 2) A 60 Hz notch filter was used to remove the line noise artifacts. 3) A band-pass filter (0.2-250 Hz) was used to filter the EEG data, with a filter steepness of 24 dB/octave. 4) Specific EEG data segments were visually inspected for artifacts associated with facial and muscular activity and were decontaminated [19]. 5) The decontaminated signals were subjected to the spatial Laplacian approach [20]. The Surface Laplacian method emphasizes sources at small spatial scales to reduce the effect of volume conduction on coherence calculations [21]. Decontaminated EEG data were used to extract the strength, search information, temporal network flexibility, integration, and recruitment features.

**Brain cortices:** Each EEG channel was assigned to a corresponding cortex based on the recording electrodes placed above each cortex [22] using the Brodmann Interactive Atlas (<http://www.fmriconsulting.com/brodmann/Interact.html>) and the Brain master software (<http://www.brainm.com/software/pubs/dg/BA_10-20_ROI_Talairach/>). Specifically, the 116 EEG channels were assigned to the four cortices.

To obtain a summary of the features for each cortex, the average of the features for channels in the frontal, parietal, occipital, and temporal cortices were calculated.

## **Extraction of search information feature**

The search information feature represents the amount of information (in bits) required to navigate the most efficient path between two nodes in the network [24].

The Adjacency matrix, commonly known as the functional brain network, of each EEG recording and the Brain Connectivity Toolbox (https://sites.google.com/site/bctnet/measures), were used to extract the search information feature [23-25]. A network that mathematically describes the connection (i.e., information transformation) strength between areas of the brain is called the adjacency matrix [26]. Entries of this matrix showed the weight of connections between various EEG channels i and j ($\text{Γ=}(\Gamma_{\text{ij}})\in\mathfrak{R}^{\text{NXN}}$; i and j ranged from 1 to N, where N is the number of EEG channels), which were determined using the coherence analysis [27]. To calculate the average search information feature at each cortex, the functional connectivity of channels in that cortex were averaged. This resulted in four average search information features.

## **Extraction of temporal network flexibility feature**

The temporal network flexibility feature is proportional to the number of times a network node changes the community it belongs to over time [29]. A network community refers to a subgroup of nodes that are more densely connected with each other than with nodes outside the community [30]. To calculate the temporal network flexibility for each channel, the flexibility function of the Network Community Toolbox (<http://commdetect.weebly.com/>) was used [33].

For each one-second window of EEG data recording, an adjacency matrix (i.e., functional brain network) was extracted in order to calculate the temporal network flexibility feature. The "community Louvain" function of the Brain Connectivity Toolbox was then used to extract the modularity metric related to each adjacency matrix. This metric measures how well nodes are assigned to communities. Modularity was maximized using a Louvain-like locally "greedy" algorithm to find the most efficient network communities [31]. This process was repeated 100 times in a consensus iterative algorithm to identify a single consistent representative partition from all partition sets, based on statistical testing in comparison to the ‘Newman– Girvan (NG)’ null network [31, 32]. The community assignment of EEG channels at each 1-second window EEG is the output of modularity maximization. The community assignment of each EEG channel is the community that the EEG channel was assigned to (e.g., each node's community assignment is an integer from 1 to 5, if five communities were found in an adjacency matrix). The partition matrix $A\in\mathfrak{R}^{\text{NXT}}$ was constructed using the community assignments of EEG channels across 1-second windows. The elements of the partition matrix $A_{i,t}\in\left\{ 1...g \right\}$ displayed the community (g) to which brain area i (EEG channels; 1 to N, where N=116) was assigned at time t (second; t=1 to T, where T denotes recording period).

The flexibility function of the Network Community Toolbox (http://commdetect.weebly.com/) [33] was then used to calculate each channel's temporal network flexibility using equation 1.

| $f_{i}=1-\frac{1}{T-1}\sum_{t=1}^{T-1} \delta(A_{i,t},A_{i,t+1})$ | (1) |
| --- | --- |

## The temporal network flexibility of channel i measures the portion of time brain area i changed its community assignment over successive 1-second time windows.

The temporal network flexibility features were calculated by averaging the flexibility scores of all channels within each cortex, resulting in 4 temporal network flexibility features. Low temporal flexibility indicates that the corresponding community assignment of each EEG channel area remains stable across time windows, while high temporal flexibility indicates that the community assignment changes frequently [29, 33].

## **Extraction of strength feature**

Functional brain network strength in a cortex refers to the degree to which different regions within that cortex are interconnected and communicate with each other [28]. It is a measure of the overall activity and connectivity within a given region of the brain. The strength of functional connectivity between different brain regions can provide important information about brain function and cognitive processes. Understanding the functional brain network strength in different cortical regions can therefore provide insight into the neural basis of cognitive processes. To calculate the average strength feature at each cortex, the functional connectivity of channels in that cortex were averaged. This resulted in four average strength features.

## **Extraction of integration and recruitment features**

Integration and recruitment features were calculated for each channel. Recruitment measures the average probability that a node belongs to the same community as other nodes from its own cortex, where each cortex was considered as a separate cortex. On the other hand, integration measures the average probability that a node belongs to the same community as nodes from other cortices [34].

Module Allegiance Matrix (MAM), which was created using the partition matrix, was used to extract integration and recruitment features. Element (i,j) in the MAM represents the probability that nodes ‘i’ and ‘j’ belong to the same community across the time windows of a recording. The interaction strength (I), between two cortices C_k1_ and C_k2_, can be defined as the average probability of pairs of channels belonging to the same community, where one electrode lies within the first cortex and the second electrode lies within the second cortex (equation 2).

| $I_{k1,k2}=\frac{\sum_{i\in C_{k1}j\in C_{k2}} P_{ij}}{\left\vert C_{k1} \right\vert\vert C_{k2}\vert}$ | (2) |
| --- | --- |

The |Ck | in equation 2 is the number of nodes in the cortex Ck, and k1 ≠ k2.

By setting k1=k2 in equation 2, the average recruitment of a single group to the task is calculated. The average integration between two separate groups is calculated using the normalized interaction between the two groups (k1 ≠ k2). Integration and recruitment features were extracted using the MAM matrix and the Network Community Toolbox (<http://commdetect.weebly.com/>) [34, 35]. The Pij in equation (2) are elements of MAM.

Finally, the average of these features across channels within each cortex was calculated, resulting in 4 integration and 4 recruitment features.
