# Supplementary material for: Performance and learning rate prediction models development in FLS and RAS surgical tasks using electroencephalogram and eye gaze data and machine learning
Source: Surg Endosc. 2023 Sep 20;37(11):8447–63. doi: 10.1007/s00464-023-10409-y (PMC10615961; doi:10.1007/s00464-023-10409-y)
Supplement: Supplementary file 2 — Supplementary file2 (DOCX 330 kb) [file 464_2023_10409_MOESM2_ESM.docx]

**Supplement 2** Representation of selecting optimum lambda value using five-fold cross-validation based on minimum Bayesian Information Criterion (BIC)

| 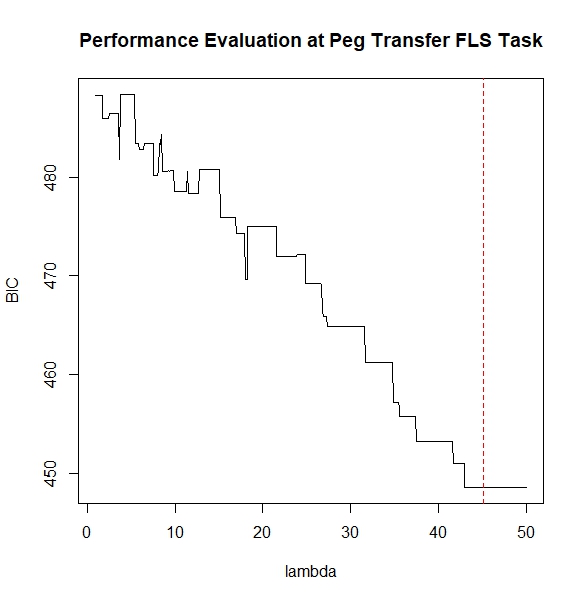  **FLS peg transfer** | **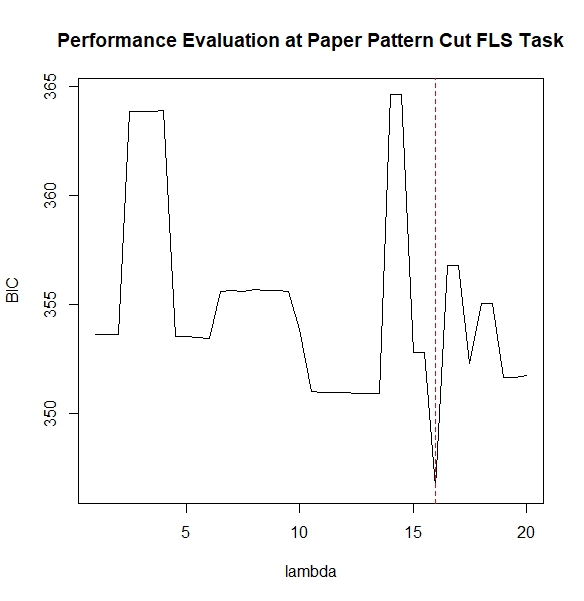**  **FLS pattern cut** |
| --- | --- |
| **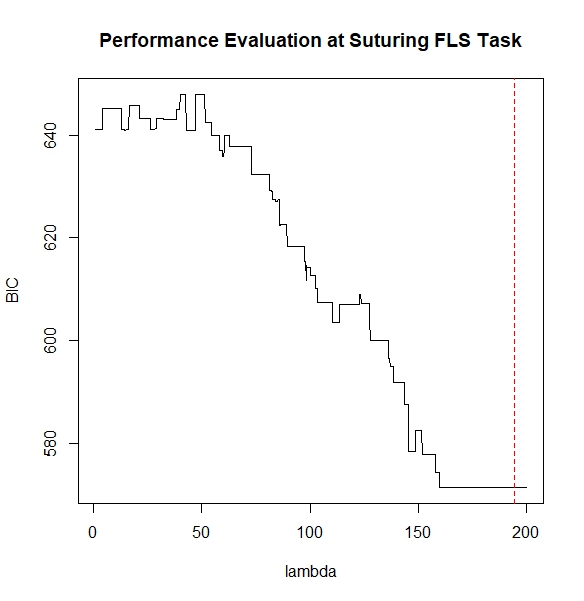**  **FLS suturing** | |
| **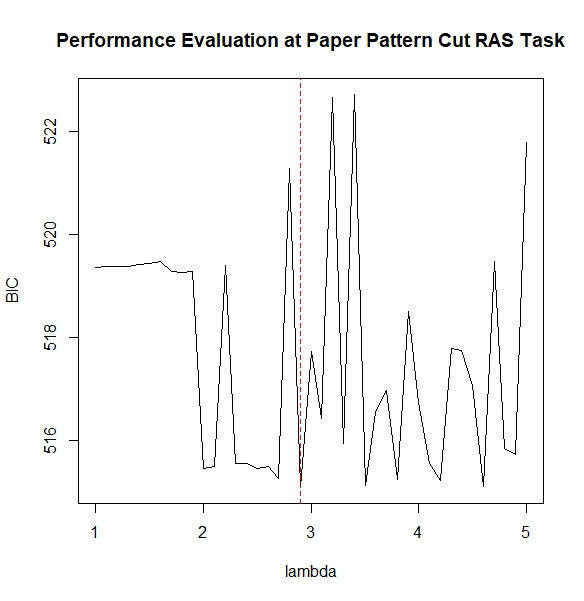**  **RAS pattern cut** | **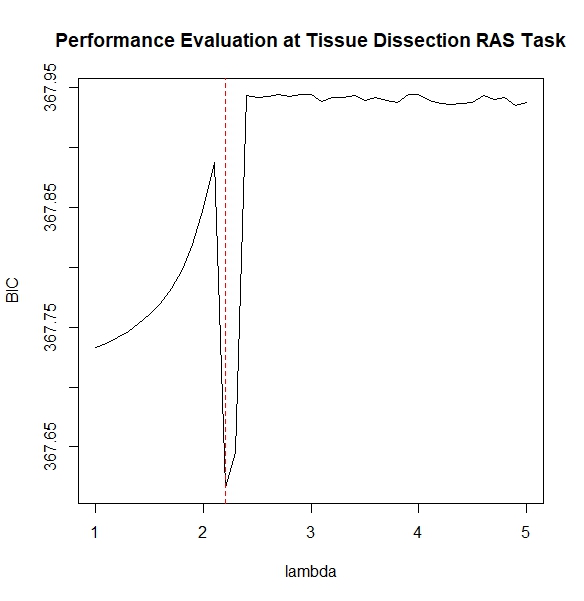**  **RAS tissue dissection** |
